# *Stretch in Focus*: 2D Inplane Cell Stretch Systems for Studies of Cardiac Mechano-Signaling

**DOI:** 10.3389/fbioe.2019.00055

**Published:** 2019-03-27

**Authors:** Oliver Friedrich, Anna-Lena Merten, Dominik Schneidereit, Yang Guo, Sebastian Schürmann, Boris Martinac

**Affiliations:** ^1^Institute of Medical Biotechnology, Friedrich-Alexander-University Erlangen-Nürnberg, Erlangen, Germany; ^2^Mechanosensory Biophysics Laboratory, Victor Chang Cardiac Research Institute, Darlinghurst, NSW, Australia; ^3^Erlangen Graduate School in Advanced Optical Technologies, Friedrich-Alexander-University Erlangen-Nürnberg, Erlangen, Germany; ^4^Muscle Research Center Erlangen, Friedrich-Alexander University Erlangen-Nürnberg, Erlangen, Germany; ^5^Faculty of Medicine, St Vincent's Clinical School, University of New South Wales, Darlinghurst, NSW, Australia

**Keywords:** mechanotransduction, mechanosensitive (MS) ion channel, cardiac mechano-electric coupling, arrhythimas, PDMS (polydimethylsiloxane)

## Abstract

Mechanobiology is a rapidly growing interdisciplinary research field, involving biophysics, molecular and cell biology, biomedical engineering, and medicine. Rapid progress has been possible due to emerging devices and tools engineered for studies of the effect of mechanical forces, such as stretch or shear force, impacting on biological cells and tissues. In response to such mechanical stimuli, cells possess various mechanosensors among which mechanosensitive ion channels are molecular transducers designed to convert mechanical stimuli into electrical and/or biochemical intracellular signals on millisecond time scales. To study their role in cellular signaling pathways, devices have been engineered that enable application of different strain protocols to cells allowing for determination of the stress-strain relationship or other, preferably optical, readouts. Frequently, these devices are mounted on fluorescence microscopes, allowing simultaneous investigation of cellular mechanotransduction processes combined with live-cell imaging. Mechanical stress in organs/tissues can be complex and multiaxial, e.g., in hollow organs, like lung alveoli, bladder, or the heart. Therefore, biomedical engineers have, in recent years, developed devices based on elastomeric membranes for application of biaxial or multiaxial stretch to 2D substrate-adhered or even 3D-embedded cells. Here, we review application of stretch technologies to cellular mechanotransduction with a focus on cardiovascular systems. We also present new results obtained by our *IsoStretcher* device to examine mechanosensitivity of adult ventricular cardiomyocytes. We show that sudden isotropic stretch of cardiomyocytes can already trigger arrhythmic Ca^2+^ transients on the single cell level.

## Introduction

The heart is an electro-mechanical organ able to transform mechanical stimuli into electrical signals (Kohl et al., 1999). The heart is pumping blood and thus, supplies organs with oxygen and nutrients. By acting at the cellular level, mechanical forces alter the cardiac electrical function in a process referred to as mechano-electric feedback (MEF). The conversion of mechanical force into electrical and biochemical intracellular signals is, e.g., mediated by mechanosensitive (MS) ion channels. To date, the molecular identity of MS ion channels underlying cardiac MEF has not been well-characterized, although several TRP-(transient receptor potential)-type ion channels have been implied in cardiac function (Ward et al., 2008; Dyachenko et al., 2009), particularly in mechano-pathologies including cardiac hypertrophy and congestive heart failure (Seo et al., 2014; Nikolova-Krstevski et al., 2017). Although there are many potential candidates among known MS channels that could underlie and contribute to cardiac MEF, there is currently no direct evidence for their role in MEF, except for the TRPC6 and TRPC3 channels (Dyachenko et al., 2009; Seo et al., 2014; Yamaguchi et al., 2017). In other cases, it remains unclear whether ion channels correlated with pathological stress responses were inherently mechanosensitive and thus, directly involved or indirectly activated by G-protein coupled receptors (Gottlieb et al., 2008; Hill-Eubanks et al., 2014; Wilson and Dryer, 2014).

Discovery of the Piezo family of MS ion channels presents one of the recent breakthroughs in eukaryotic mechanobiology (Coste et al., 2010). Given the recent evidence showing the important role that Piezo1 mechanosensitive channels play in cardiovascular mechanosensing (Li et al., 2014), the underlying molecular mechanisms have attracted growing interest, including further studies of the respective mechanosensors in cardiac signaling, i.e., MEF, and their associated signaling pathways. To allow direct investigation of the mechanosensory signaling *in vitro* by applying stretch or shear forces to cardiomyocytes and cardiac or vascular endothelial cells, it is important to employ devices for application of different mechanical strain protocols mimicking as close as possible those experienced by cardiac and vascular cells *in vivo*. Such investigations should also help to reconcile previous correlative studies of ion channel expression and function under conditions of heart disease with single cell models (Friedrich et al., 2012, 2017). Hemodynamic volume/pressure load in the heart, as a hollow organ, is associated with multiaxial wall distension. A volume/pressure overload causes *inplane* 2D stretching of individual cardiomyocytes in multiple directions (Friedrich et al., 2017). This challenge prompted biomedical engineers to design and further develop multiaxial cell stretch systems, which have enabled studies of chronic heart distension on a cellular level.

**Figure 1:**
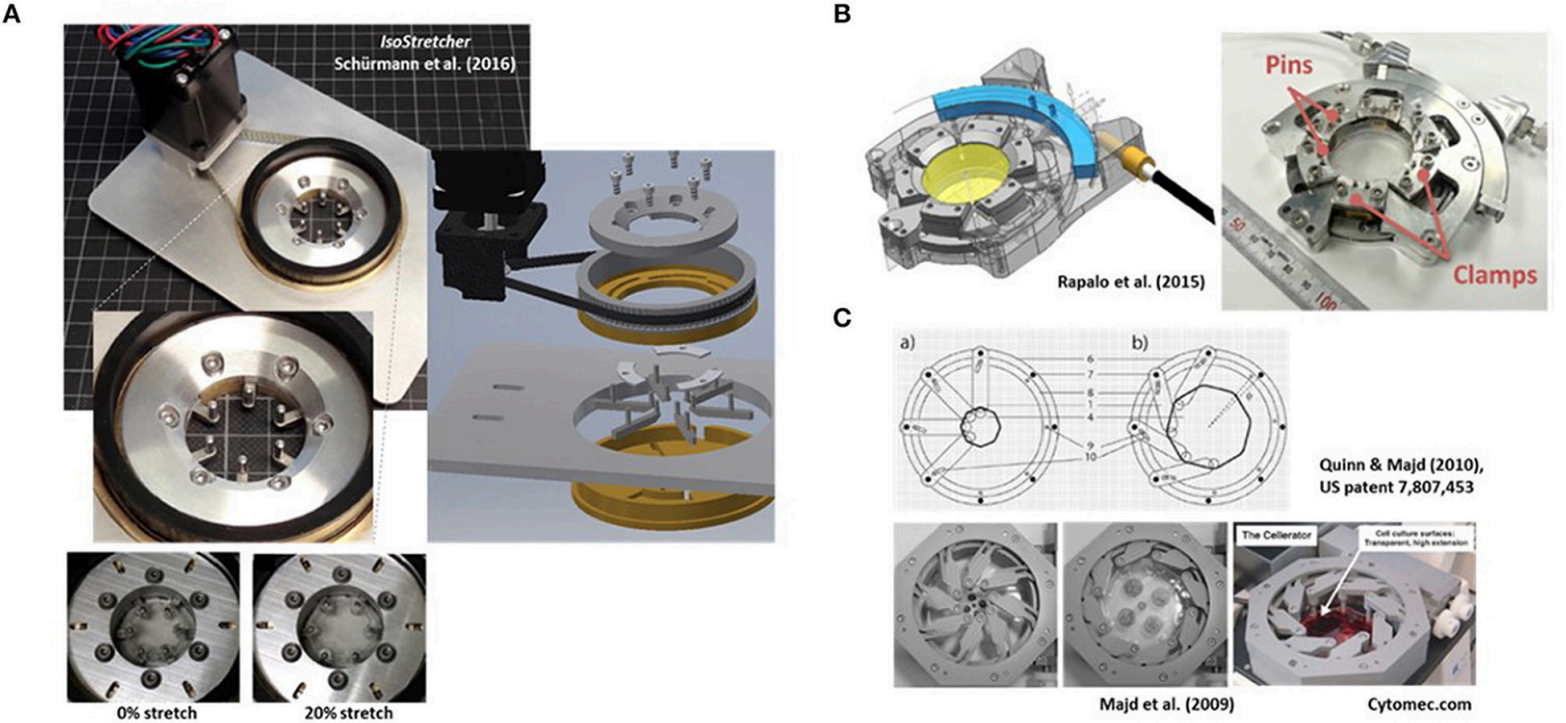
Examples of research-built and—inspired commercial inplane stretch systems that can be suitable for studies of cardiac cells and mechano-electric feedback. **(A)** The *IsoStretcher* device, first described in Schürmann et al. (2016) that uses a V-belt driven, swivel motor actuated rotational-to-radial translation for displacement of six hook-sliders to stretch a PDMS-cast biochamber. Maximum radial stretch of the system is ~20%. An improved current version (2018) is shown. **(B)** Radial displacement PDMS chamber lip clamp system introduced by Rapalo et al. (2015) to combine isotropic stretch of cells in large culture dishes (about 4 cm diameter) for confocal of atomic force microscopy. Maximum linear strain was 20%. Taken with permission from Rapalo et al. (2015). **(C)** An iris-like actuated system that uses eight PDMS substrate (HERS: high-extension silicon rubber) holding arms that are screwed to an outer frame allowing rotational degree of freedom of movement while the inner substrate pillar will be pulled toward the outer frame once the outer ring is actuated. For details see Quinn and Majd (2010). Isotropic surface expansions up to 1,000% have been described (Majd et al., 2009). The system was commercialized as Cellerator by the Swiss company Cytomec until 2017. (Adapted from Majd et al., 2009).

In this paper, we briefly review recent approaches in biomedical engineering toward development of stretch devices enabling application of biaxial or multiaxial stretch to cells. We further discuss the advantages of the *IsoStretcher* (Figure 1A), a new cell stretch system engineered by the authors that overcomes some previous limitations (Schürmann et al., 2016). In addition, we show that single adult ventricular cardiomyocytes can be stretched isotropically when following a 3D-hydrogel embedding approach that allows for inplane cell stretch to be applied and Ca^2+^ transient activity to be immediately observed with minimum z-shift of the optical axis.

## Pulling the Strings and Beyond

Stretching single cells can be a tedious and cumbersome undertaking, in particular with smaller cell geometries. Given their large sizes with diameters up to ~100 μm and lengths from several hundreds of μm up to exceeding 10 cm, depending on the species, skeletal muscle single fibers have been a first prototype of cells subjected to longitudinal stretch. Since skeletal muscle serves predominantly as a linear bioactuator, uniaxial stretch systems were the obvious design. Early systems were research-designed machines, mostly consisting of an opposing configuration of a force transducer pin and a static counter-pin of infinite stiffness, the latter of which could be actuated to stretch the preparation, while the former served to measure passive restoration forces and/or active force generation upon fiber activation (e.g., Ter Keurs et al., 1978; Moss, 1979). The dissection, handling, and fixing of single muscle fibers into such biomechatronics systems for subsequent biomechanics recordings still is a tedious procedure confined to a few labs worldwide with also limited throughput due to manual handling of the systems (Lamb and Stephenson, 2018). As for their lengths, single muscle fibers can well be manually tied to macroscopic needles for stretching using micro-knots from braided silk or tweezer clamps with a long portion of the single cell preparation still intact between the stretch posts (Roche et al., 2015). Recently, such a biomechatronics system capable of directly assessing stress-strain relationships has been automated in a robotized environment to increase throughput (Haug et al., 2018).

However, in cases where cell types become substantially smaller than skeletal muscle fibers, the concept of manually tying/clamping cells to stretch posts/needles becomes non-feasible. This applies to most other cellular systems of the body where cells mostly fall into the range of tens of microns up to just exceeding 100 μm. For rod-shaped human ventricular cardiomyocytes that are at the upper end of the spectrum, typical diameters are ~25 μm and lengths range 60–140 μm (Tracey and Sander, 2011). Bioengineers around Jonathan Lederer from Fischell Department of Bioengineering of University of Maryland drove the engineering and validation of a so-called *Cell Tester* device, together with a small enterprise from Heidelberg, Germany (Scientific Instruments, SI Heidelberg). This system that is now commercialized by World Precision Instruments (https://www.wpiinc.com/blog/category/cell-tester/) enables to manually grab individual cardiomyocytes by their ends and stretch them uniaxially. The system contains a chamber holding a 35 mm glass bottom dish containing, e.g., cardiomyocytes, and a rotational cuvette around the dish rim to also apply jets of solution to facilitate positioning of a single CM in the XY plane with their long axis in between an optical force transducer pin and an actuator-pin connected to a stepper motor for stretching cells. An electrically-driven clamp-tweezer mechanism allows one to grab and squeeze both ends of a single cell tightly. The whole system fits on top of inverted research-microscopes to perform, for instance, fluorescence recordings. The optical path of the microscope remains undisturbed and z-focus is adjusted with actuating the focus lens. Applying 8% uniaxial stretch to single cardiomyocytes and recording Fluo-4 Ca^2+^ sparks, Prosser et al. were able to identify a “stretch-induced tuning of RyR2 to increase Ca^2+^ signaling sensitivity in healthy cardiomyocytes” and trigger Ca^2+^ sparks in a nicotinamide adenine nucleotide phosphate oxidase 2 (NOX2)-reactive oxygen species (ROS)-dependent process (Prosser et al., 2011). Although an elegant bioengineering solution to an emerging problem of studying mechano-chemical coupling in the heart, the system has several limitations: (i) it is bulky and represents a major investment (roughly 50 kUSD), (ii) through squeezing the cells at the end, parts of the cell may be strongly damaged, (iii) it can only be used for acute or short-term observations, investigating either freshly bioseparated or cultured cardiomyocytes/cells (i.e., it has no bioreactor chamber), (iv) handling is still limited to one cell at a time thus, limiting throughput and moreover, (v) cells are investigated in a free-floating environment void of any cell-cell contact or extracellular matrix which is important when addressing questions involving focal adhesion complex (FAC) regulation, and (vi) stretch is purely uniaxial. Although some points can be worked around, e.g., for (ii) using a bioadhesive glue to attach cells (Prosser et al., 2011) or (v) by using organoids, the limitations of throughput, restriction to uniaxial stretch and unavailability of FACs in single cells are of a system-inherent nature.

## 2D Inplane Cell Stretch Systems for High-Content Microscopy

In order to increase throughput in so-called high-content assays, engineers have explored alternative ways of developing silicon-elastomer-based methodologies to allow adherence of many cells on flexible substrates for defined stretches to cells. A detailed review of the history of poly-dimethyl-siloxane (PDMS) polymer-engineering and its properties related to biocompatibility, elasticity and hydrophobicity can be found in our previous work (Friedrich et al., 2017). Briefly, PDMS is highly biocompatible and bioinert, and its elasticity can be tuned by varying the ratios of base DMS compound and cross-linker before polymerization. Due to its high hydrophobicity, it must be functionalized prior to seeding and attachment of cells (Friedrich et al., 2017). With tuning of the substrate elasticity, the stiffness of respective tissues can be mimicked in order to facilitate FAC building of seeded cells simulating their natural environment (i.e., soft vs. stiff substrates). However, for matrices mimicking elasticity moduli of very soft tissues with values of ~1 kPa or below (e.g., stem cells, neuronal tissue; Even-Ram et al., 2006), either use of polyacrylamide gels or PDMS blends (using commercially available such as Sylgard 527 and 184) has been shown to be superior over single PDMS types (Palchesko et al., 2012). Including these environmental mechanical cues into cell culture technologies has become an indispensable tool in mechanobiology (Engler et al., 2006; Kurpinski and Li, 2007; Wipff et al., 2009), and also for cardiac research (Galie et al., 2013). Using downstream chemical processing of custom-made PDMS membrane geometries after curing in molds, extracellular matrix proteins, e.g., collagen, fibronectin, laminin, etc., can be covalently cross-linked to the stretchable PDMS substrate following PDMS oxygenation and silanization to increase hydrophilicity, which significantly improves attachment, spreading and proliferation of, e.g., fibroblasts (Wipff et al., 2009).

The major challenge in applying strain to PDMS membranes containing an adhered cell system is to define the directionality of stretch regarding the strain axis to be actuated and the respective biological readout for the respective cell system. For a long time, pneumatically driven systems were the leading technology, commercialized e.g., by FlexCell International Corporation (http://www.flexcellint.com/). This included sealing the PDMS membrane against a closed chamber to which negative or positive pressure could be applied via an external pressure generator. Obviously, the bulging of the membrane, although allowing for extended cyclic stretch trains, precluded use of imaging due to vast focus shifts of the substrate membrane (e.g., Kreutzer et al., 2014). A detailed discussion of those systems is given in Friedrich et al. (2017).

In order to pursue bioengineering of stretchable substrates for a more *inplane* stretch suitable for simultaneous microscopy, uniaxial stretch systems were developed as the predominant mode of actuation at the time. Those PDMS chambers were slid over polymer or metal rods on the outer chamber rim, fixing them to the base plate of a stepper motor geometry for strain applications and mounted on inverted microscopes. Using such an approach for 2D strain-culture of endothelial (HUVEC, human umbilical vein endothelial cells) cells, a preferential alignment of cells perpendicular to the main strain axis was observed (Matsumoto et al., 2007). This was also confirmed in our recent studies using atrial endothelial cells (Nikolova-Krstevski et al., 2017). Applied to endothelial cells in 3D, uniaxial strain direction was found to regulate directionality of cellular process sprouting within the hydrogel (fibrin-gel) (Matsumoto et al., 2007). In another study focusing on human bone osteosarcoma cells, a custom-made stretch device applying 5% uniaxial stretches to 50 kPa stiff elastic silicone films to which cells were adhered via fibronectin-coating was able to demonstrate rapid focal adhesion growth within seconds after stretching (Chen et al., 2013). All those biological processes were accessible to live-imaging, proving the inplane stretch criterion for associated imaging. However, one must keep in mind that z-focus shifts are inevitable due to volume conservation considerations of the material upon stretch in the elastic deformability regime thus, with stretch, the substrate membrane will always become thinner and the focus eventually shift. Although uniaxial PDMS substrate stretch systems suitable for reproducible cyclic stretch and live cell imaging have been employed, for instance, to visualize YFP-paxilin FAC remodeling in rat embryonic fibroblasts, the thinning of PDMS membranes in simple clamp-stretch devices usually requires manual re-adjustment of focus before acquiring cell images after each stretch (Shao et al., 2013). As detailed below, designing a chamber geometry with adequate mass distribution on the walls to dissipate the strain in order to minimize focal shifts of the thin membrane window portion has become the most challenging engineering aspect which becomes more eminent with higher optical resolution imaging techniques. One of the current market leaders in distributing PDMS stretch chambers with actuators for uniaxial stretch applications is STREX Inc. in Osaka, Japan (http://strex.co.jp/).

## Indenter Ring-Based Inplane Cell Stretch Systems With Flexible Strain Geometries

In the last few years, biophysical considerations regarding wall tension in hollow organs have led to a refinement of more physiological requirements toward strain applied to cellular systems (Huang et al., 2010; Friedrich et al., 2012). It was hypothesized that physiological wall tension at least was multiaxial in most cases, and even equibiaxial or isotropic in some case, for instance lung alveoli, bladder urothelia, etc. (Arold et al., 2007; Friedrich et al., 2012). Also, for more complex hollow organs like the heart, equibiaxial stretch during diastole might be a first approximation, while in skeletal muscle as linear bioactuator, uniaxial strain certainly remains the primary mechanical stressor. With these considerations came the necessity for new bioengineered systems to routinely apply multiaxial stretch to cells on flexible elastomer membranes while additionally allow for high-resolution microscopy with minimum focal shift. The very first systems employed the concept of actuating indenter rings over the PDMS membrane up and down an underneath indentation post to stretch and de-stretch the membrane as the indenter pulled the membrane down and up, respectively (Hung and Williams, 1994; Sotoudeh et al., 1998). By employing indenter rings with either central circular or rectangular geometry, Huang et al. (2010) were able to switch between uniaxial and equibiaxial (isotropic) stretch by simply exchanging the indenter rings. Mounting this system on top of an inverted microscope, they were able to show that equibiaxial (isotropic) stretch induced more focal adhesion complexes between cells and fibronectin-coated PDMS substrate when applying biaxial over uniaxial cyclic stretch in a 2D culture of bovine aortic endothelial cells, demonstrating the differential effects of both stretch regimes (Huang et al., 2010). By inclusion of tracking particles in the PDMS layer of known substrate stiffness, traction force microscopy can even be applied by evaluating the displacement distribution of tracking particles and calculating local strain fields (Wipff et al., 2009; Legant et al., 2010). Although equibiaxial stretch systems had been refined in the last few years for strain homogeneity (Urseka et al., 2014), those systems have to our knowledge largely remained confined to the academic community with no larger attempts for commercialization. One shortcoming of indenter-based systems is in the permanent direct contact of the PDMS membrane with the post material. This can readily lead to elastomer damage and pre-mature rupture in long-term tests. Some reports have mentioned use of lubricants between loading posts and PDMS membrane to reduce friction (Kreutzer et al., 2014) which, however, may also limit visualization attempts. Given those considerations, a free-floating substrate, rather than a direct contact configuration through indentation of the PDMS membrane, might be preferable.

In order to follow such an approach, we and others have bioengineered isotropic stretch systems based on either radial displacement of point-fixations on the outer periphery of a circular stretch-chamber (Rapalo et al., 2015; Schürmann et al., 2016) or an iris-like mechanism (Majd et al., 2009). Those will be the focus of the following sections, followed by new application data from our *IsoStretcher* system to ventricular cardiomyocytes. One pneumatically-driven equibiaxial stretch system containing elastomeric PDMS micropost arrays suitable to convert pneumatically controlled negative pressure to bending of microposts and thus, traction forces on point attachments to cell membranes in a lab-on-a-chip format for high content imaging, shall be mentioned here for completeness (Mann et al., 2012).

## Radial Displacement Actuation Technologies (e.g. *IsoStretcher*)

In 2016, we described the first generation of the *IsoStretcher*, an inplane isotropic stretch system. This employs equitriaxial radial displacement of a circular PDMS membrane-designed stretch chamber by a V-belt translated, grab swivel motor-driven radial displacement of six evenly distributed pull points in the periphery of the chamber through six linear sliders (Schürmann et al., 2016). Those sliders are guided in six radially oriented grooves underneath the chamber drilled into the lower base with two upward-facing pins at each end. One end is inserted into equivalent holes of the PDMS chamber ring while the pin of the outer end is inserted into a translation ring connected to the V-belt drive, containing six oblique grooves to guide the pins to the outer radial position as the ring turns. Figure 1A shows an improved current version of the system, reflecting a market prototype for upcoming commercialization. Compared to the previous version (Schürmann et al., 2016), polymer materials in moving parts have been replaced by steel and aluminum parts for better durability, the microcontroller and software updated and PDMS chambers refined for larger volumes of up to 1 ml as compared with the previous low volume chamber of ~100 μl. New casting molds were also designed and polished, resulting in better transparency of the PDMS bottom for microscopy. We have validated the system to prove isotropicity and homogeneity of stretch as well as confirming a very low z-drift during stretch in the range of ~15 μm under optimum conditions, allowing one to follow cells during stretch in real time (see supplemental video in Schürmann et al., 2016). One conclusion from our previous study was that increase in cell surface area had to be calibrated once for each new cell line and coating combinations to make sure that cells actually follow the applied hardware stretch and did not (partially) detach from the substrate, giving rise to false interpretations (Schürmann et al., 2016). Unlike in uniaxial stretch where the sample stretch matches the hardware stretch, in isotropic systems, the percentage increase in radial displacement dr/r translates to the PDMS substrate area increase dA/A according to:

(1) $$\begin{array}{l} dA=2\pi \cdot r\cdot dr=2\pi \cdot \frac{r^{2}}{r}\cdot dr\;\Leftrightarrow \;\frac{dA}{A}=2\cdot \frac{dr}{r} \end{array}$$

This means that a 10% increase in membrane area is achieved by a 5% radial pin displacement in the *IsoStretcher*. The IsoStretcher comes with a base plate to fit the stage of any commercial inverted research microscope and allows excellent high content imaging with long working distance objectives (modifications toward high-resolution immersion imaging are possible). The system is very light (~200 g) and allows hardware stretch up to 20% (membrane stretch of 40%). The system allows one to apply cyclic and static stretch application. The stretch profile can be programmed to follow any given profile (saw tooth, sinusoidal, or rectangular). Given the minimum displacement velocity of 0.1%/s, and maximum around 40%/s, we usually use 20%/s which allows a 20% stretch within a second. Velocity can be tuned in steps of 0.1%/s, where the smallest displacement resolution is at 0.05% radial displacement.

At about the same time, Rapalo et al. (2015) presented a similar, yet more bulky system, to isotropically stretch PDMS membrane chambers of ~30 mm diameter using six evenly spaced clamp tabs containing two holes for fixation to the posts of six clamps (Figure 1B). A linear driver converts the motor rotation to a one-dimensional motion to control isotropic stretch of up to 20% radial displacement of clamps (Rapalo et al., 2015). Translation of radial strain to linear displacement between human bronchial epithelial cells (16HBE) cultured directly on the PDMS membrane was verified by measuring the nearest neighbor distances of fluorescently labeled (DAPI) nuclei (Rapalo et al., 2015). However, in contrast to our approach of directly measuring cell surface extension upon stretch and thus, dA/A, no affirmation of whether cells were indeed tightly adhered to the PDMS membrane was provided. Also, the report did not state any functionalization of the PDMS membrane with matrix proteins or oxygenation, which renders a tight adherence to the hydrophobic PDMS membrane questionable. So far, there is no update on further refinement of the system, nor its commercialization.

## Iris-Like Stretch Device Systems for Large Area Increase Applications (*Cellerator*)

In 2009, Majd and colleagues from the Hinz lab at EPFL, Lausanne, published a novel system for dynamic cell culture of stem cells under isotropic stretch (Majd et al., 2009), based on their US patent US 7,807, 453 B2 (issued October 5, 2010) “Device for cell culture on deformable surfaces” (Figure 1C). High-extension silicon rubber (HERS; elastic modulus 8–25 kPa, Majd et al., 2009) was injection-molded in the shape of a cell culture dish containing elastomer hollow pillars fitting around the periphery of the chamber. Those were slid over pins within an iris-like actuated system with eight holding arms screwed to an outer frame allowing rotational movement thus, pulling the inner substrate pillars toward the outer frame (Figure 1C). Using the HERS over conventional PDMS, surface expansions of up to 1,000% were described. Those were used in confluent 2D cell cultures to maintain relatively constant cell densities during proliferation and to prevent contact inhibition. Since the HERS dish was biocompatible and of very good transparent quality, it was used to keep human mesenchymal stem cells over 9 weeks in dynamic expansion culture without contact inhibition and under optical surveillance (Majd et al., 2009). The system was further used in subsequent studies by the inventors (Majd et al., 2011; Khayat et al., 2012; Klingberg et al., 2014) and was commercialized as *Cellerator* device by the Swiss company Cytomec, founded by T.M. Quinn in 2005, until 2017 when the company was dissolved.

In summary, isotropic or biaxial stretch devices for mechanobiology research have not been widely introduced on the market for a broad audience, and with the cessation of Cytomec and the *Cellerator*, the only other company with a biaxial stretcher portfolio seems to be *CellScale Biomaterials Testing* (www.cellscale.com/products/mcb1/). Their system uses a sophisticated star-shaped mesh pulling eight connected pillars holding a PDMS dish underneath. However, since their system is enclosed in a bioreactor box with no optical access, there is currently no other commercial solution available for live-cell imaging of stretched cells. To fill the gap, attempts for commercialization of the *IsoStretcher* are underway.

## Application of the *IsoStretcher* in Heart Mechanobiology Research

In our initial publication, we had validated the *IsoStretcher* for reliable inplane stretch of 2D cell cultures using endothelial or epithelial cell lines (e.g., HeLa cells, HEK293 cells, atrial HL-1 cells; Schürmann et al., 2016; Friedrich et al., 2017). Since then, we have applied our system to other adherent cell lines in ongoing studies. In our hands, with the right ECM protein coating, endothelial and epithelial cells readily and tightly adhere to the substrate for microscopy assessment during isotropic stretch. As detailed in Friedrich et al. (2017), when turning to adult ventricular cardiomyocytes, we could not find any matrix-functionalization procedure allowing for tight adherence of CMs in 2D culture. Inspired by a CM “cell-in-a-gel” approach (Jian et al., 2014), we experimented with 3D-hydrogel embedding of adult CM and found polyvinyl-alcohol (PVA) hydrogels, doped with thiol groups to tune their matrix stiffness for CM ECM to be a feasible bioprocess approach (Friedrich et al., 2017). In that previous report of ours, we demonstrated reliable membrane area increase upon stretch using the *IsoStretcher* up to a linear hardware stretch of 15% in medium to strong gels containing 5–9 mM thiol groups (Friedrich et al., 2017). Stretching the gel was accompanied by a stretch-induced Ca^2+^ entry into CMs from the external bulk media, as visualized by confocal Fluo-4 Ca^2+^ fluorescence microscopy. Since this increase in fluorescence developed over a time course of several minutes, which is unusually slow for live-cell reactions, the only remaining explanation could be seen in a vast diffusion restriction even to small molecules through the PVA-hydrogel. Consequently, we revisited this hypothesis to provide experimental evidence.

**Figure 2:**
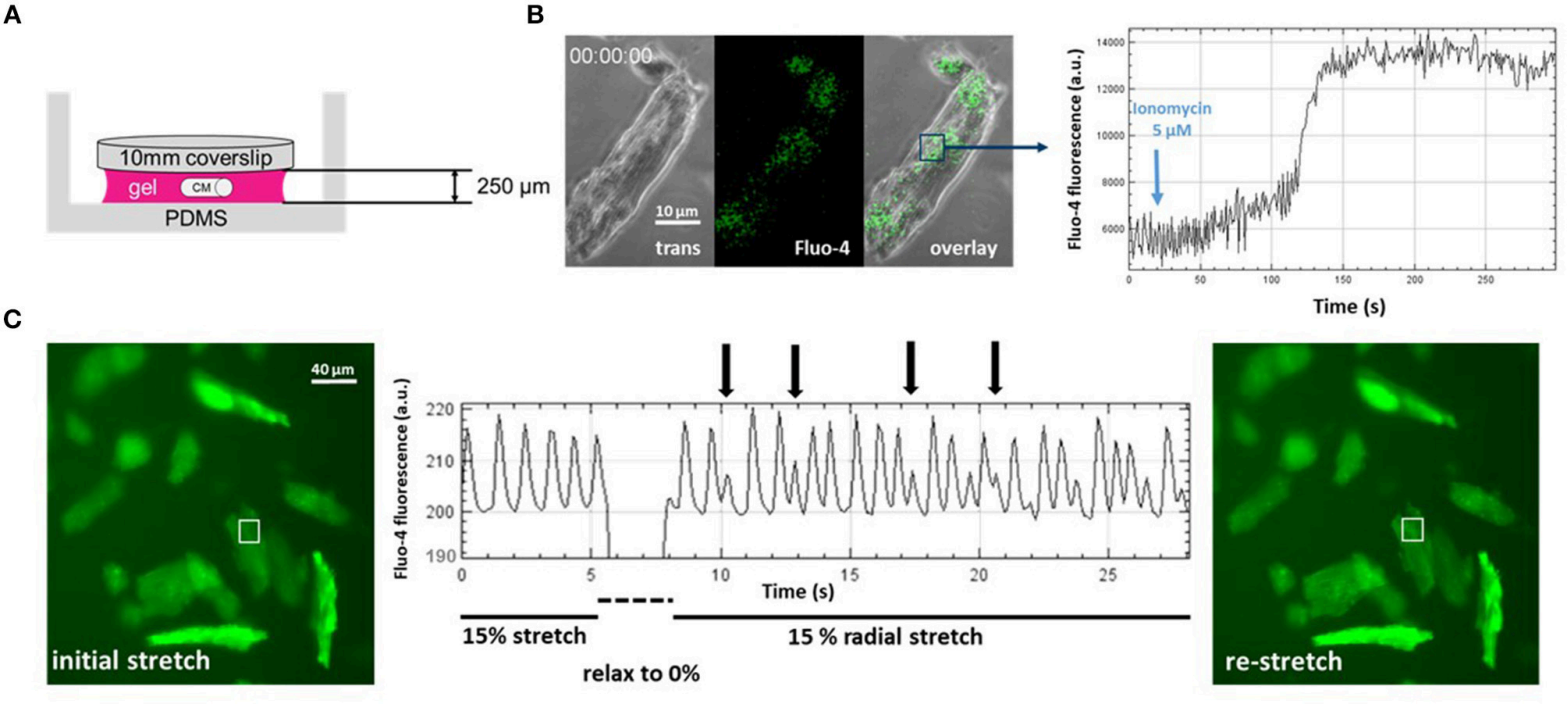
Direct visualization of mechanoelectric feedback in cardiomyocytes through *IsoStretcher* technology. **(A)** Thin polyvinyl-alcohol gel embedding of adult cardiomyocytes allows diffusion-limited accessibility to pharmacological manipulations as shown for application of a Ca^2+^ ionophore (5 μM ionomycin) to the external solution and visualization of a maximum Fluo-4 response after ~150 s **(B)**. **(C)** Proof-of-concept recording demonstrating mechanoelectric feedback, i.e., the direct visualization of mechanical isotropic stretch (15% radial stretch) inducing early after- depolarization spontaneous Ca^2+^ transients (vertical arrows) upon sudden re-stretch from the relaxed state. Note that the dip in fluorescence reading during the brief relaxation is mostly due to the radial displacement of the respective cardiomyocytes out of the ROI, which however, is perfectly restored upon re-stretch.

First, we further optimized the hydrogel layer thickness required for reliable embedding to about 10 times the CM diameter (Figure 2A). When applying 5 μM ionomycin, a selective Ca^2+^ ionophore, to the bulk solution and monitoring Fluo-4 fluorescence in stained single CMs embedded in the gel, we could reduce the pharmacological action delay down to ~2.5 min, as seen by the delay in steep fluorescence increase following the addition of the ionophore (Figure 2B). Thus, unlike in single cell experiments in open dishes where cell reactions are almost instantaneous, in hydrogel-embedded CMs, care needs to be taken to allow for sufficient recording time to catch the delay in drug delivery due to diffusional barriers for the applied drugs to reach their target (Amsden, 1998), here ionomycin. In a final step, by applying sudden de-stretch and re-stretch to PVA-embedded, Fluo-4 stained intact murine adult ventricular CMs, we could visualize stretch-induced arrhythmic Ca^2+^ transient activity online using our *IsoStretcher* device (Figure 2C). This represents the very first direct visualization of stretch-induced early after-depolarization activity of ventricular CM Ca^2+^ transients and demonstrates mechano-electric feedback on a single cellular level.

## Conclusions

Our understanding of mechano-related cellular events, particularly for hollow organs, relies on novel biomechatronics technologies that mimic the wall strain profiles of the natural tissues as closely as possible. In that regard, it became apparent that uni- vs. multiaxial strains are answered by markedly different FAC remodeling patterns. Isotropic or multiaxial stretch systems are not readily available on the market for a broad user community, and so far, biomedical engineering-inspired devices have mainly evolved at research institutions. Our *IsoStretcher* allows easy-to-use and intuitive handling alongside with many cell types, either in 2D or even in 3D cultures. With adult cardiomyocytes as one of the probably most cumbersome cell types for isotropic stretch, we succeeded to employ a PVA-hydrogel-based embedding and stretch approach that allows online Ca^2+^ imaging of cellular reactions. In doing so, we could provide the first evidence of direct visualization of mechanoelectric feedback in a mechanically-actuated 3D environment. In future studies, we aim to unravel the molecular origin of the mechanosensors(s) and channels involved but we already suspect a strong candidate: Piezo1.

## Materials and Methods

### Generation of Thin Gels

Murine ventricular cardiomyocytes, dissociated from adult C57BL/6 (90 d) mice in a Langendorff preparation were obtained through tissue sharing with other groups at the Victor Chang Cardiac Research Institute (institutional approval number: AEC 17-17). CMs were suspended within the uncured PEG-PVA gel precursor (recipe see below) and a 15 μl droplet was placed on the surface of an *IsoStretcher* PDMS chamber. A standard 0.15 mm thick glass coverslip with a diameter of 10 mm was placed on top of the droplet, generating a fluid layer, about 250 μm thick, between slide and chamber by forming the equilibrium between gravitational and capillary forces. After curing the PEG-PVA gel for 20 min, the chamber was filled with 300 μl cell culture medium. The glass coverslip was removed with forceps, leaving behind CMs embedded in an even hydrogel with defined height. The hydrogel height was determined with a confocal microscope by focusing on the clearly visible upper and lower surfaces of the gel.

### Hydrogel Recipe

Hydrogels were prepared from *Cellendes 3-D Life PVA-PEG Slow Gelling Hydrogel kits* (G82-1). The applied recipe is listed in Table 1. The components were added in sequence as they are listed in the table from top to bottom. After adding the RGD peptides, the mixture was incubated for 30 min at 37°C to allow for annealing of the peptides to the PVA thiol groups. When adding cell and PEG-Link crosslinker, the mixture was firm enough to be touched or covered by liquid without disintegrating after an incubation time of 20 min at 37°C.

**Table 1:** Cellendes 3-D Life PVA-PEG hydrogel recipe for a gel containing 4.5 μM cross-linked thiol-groups and 0.5 μM RGD peptides.

**30 μl Hydrogel med 4.5**	
Water	10 μl
10 × CB	2.5 μl
PVA	5 μl
RGD	0.75 μl
Cell suspension	5 μl
PEG-Link	6.75 μl

*CB buffer is a part of the G82-1 kit from Cellendes*.

### Determination of Diffusion Accessibility of Embedded Cardiomyocytes

Fluo-4 loading of CMs was prepared in a hydrogel of ~250 μm thickness. The gel was covered with 100 μl medium containing 3 μM Fluo-4 AM and incubated for 2 h at 37°C and 5% CO_2_. The Fluo-4 loaded (DMEM was used as cell culture medium) cells in a hydrogel were mounted into the *IsoStretcher* and imaged with a confocal microscope (Zeiss LSM 700 Inverted) using a 488 nm laser source as illumination for the fluorescence channel, while simultaneously recording a phase contrast image. A short-pass filter with a cut-off at 540 nm as well as a 488 nm notch filter were used to separate excitation from emission light. Videos with a frame time of 600 ms (512 × 512 px; 0.63 × 0.63 μm voxel size) were recorded. In the experiment shown in Figure 2B, the sample was stretched to 10% radial stretch and 20 s after starting a video recording, ionomycin was added into the chamber to a final concentration of 5 μM. The fluorescence intensity of an ROI in the cell is tracked, allowing one to visualize Ca^2+^ fluorescence intensity as well as the time point of terminal contracture of the cell.

### Assessment of Mechanoelectric Feedback in Adult 3D-Embedded CMs

Hydrogel embedded adult murine ventricular CMs were loaded with Fluo-4 in an *IsoStretcher* chamber and mounted with the *Isostretcher* on an epifluorescence microscope. Instead of cell culture medium, the hydrogel was covered with 400 μl HBSS (Hank's Balanced Salt Solution; Thermo Fisher) solution. Fluorescence was excited by a broad band UV-source and emission light and separated by a 558 nm bandpass filter. Image sequences were recorded with a frame time of 110 ms (2,048 × 2,048; voxel size 0.59 × 0.59 μm). The chamber was stretched to 15% radial stretch and the cells were allowed to adapt to the stretched environment for 5 min. A video recording was started and after 5 s of recording, the chamber suddenly relaxed to 0% and re-stretched to 15% radial stretch within 2 s. Spontaneous calcium transients of recorded cardiomyocytes were visualized by plotting the mean fluorescence intensity of a 10 × 10 μm ROI on a cardiomyocyte.

## Data Availability

The datasets generated for this study are available on request to the corresponding author.

## Author Contributions

BM and OF designed and supervised the project. A-LM, DS, and YG conducted all IsoStretcher experiments and data analysis. SS made and DS wrote the software for the IsoStretcher device. All authors discussed the results and contributed to writing of the manuscript.

### Conflict of Interest Statement

The authors declare that the research was conducted in the absence of any commercial or financial relationships that could be construed as a potential conflict of interest.
